# Rabies Reemergence, Central Europe, 2022–2024

**DOI:** 10.3201/eid3202.251597

**Published:** 2026-02

**Authors:** Emmanuelle Robardet, Marcin Smreczak, Anna Orłowska, Peter Malik, Alexandra Nándori, Zuzana Dirbáková, Rastislav Odaloš, Oleksii Rudoi, Ivan Polupan, Oxana Groza, Serghei Arseniev, Florica Barbuceanu, Vlad Vuta, Evelyne Picard-Meyer

**Affiliations:** ANSES, Nancy Laboratory for Rabies and Wildlife, European Union Reference Laboratory for Rabies, Bâtiment H, Technopôle Agricole et Vétérinaire, Malzéville, France (E. Robardet, E. Picard-Meyer); National Veterinary Research Institute, National Reference Laboratory for Rabies, Pulawy, Poland (M. Smreczak, A. Orłowska); National Food Chain Safety Office Veterinary Diagnostic Directorate, National Reference Laboratory for Rabies, Budapest, Hungary (P. Malik, A. Nándori); State Veterinary and Food Institute, Veterinary Institute Zvolen, Zvolen, Slovakia (Z. Dirbáková, R. Odaloš); The State Scientific and Research Institute of Laboratory Diagnostics and Veterinary Sanitary Expertise, Kyiv, Ukraine (O. Rudoï, I. Polupan); Republican Centre for Veterinary Diagnoses, Chisinau, Moldova (O. Grozda, S. Arseniev); Faculty of Veterinary Medicine, Splaiul Independentei, Bucharest (F. Barbuceanu); Institute for Diagnosis and Animal Health, National Reference Laboratory for Rabies and World Organisation for Animal Health Reference Laboratory for Rabies, Bucharest, Romania (F. Barbuceanu, V. Vuta)

**Keywords:** Rabies, viruses, zoonoses, sylvatic, lyssavirus, fox, oral vaccination, variant, Europe

## Abstract

Oral rabies vaccination campaigns helped eliminate rabies from parts of Europe, but rabies appears to be reemerging. We analyzed 2022–2024 data, which demonstrated reemergence of 2 virus variants; both were detected in Ukraine, Moldova, Poland, and Romania. Our findings highlight the need to strengthen rabies control efforts in the region.

Rabies had been eliminated from Western and Central Europe through regular implementation of oral rabies vaccination (ORV) campaigns among red foxes (*Vulpes vulpes*), the primary disease reservoir (*1*). Those campaigns, initiated over large and continuous areas since the 1990s, were typically conducted twice a year, in spring and autumn, and used an average bait density of 20–25 baits/km^2^ (*1*). 

The year 2020 was considered pivotal in the elimination of the classic *Lyssavirus rabies* virus (RABV) because, until that date, only sporadic cases were detected at the eastern border of the European Union in the previous 3 years (*1*). The European Commission Animal Disease Information System (https://webgate.ec.europa.eu/tracesnt/adis/public/notification) reported 8 cases/year in 2017 and 2018, mainly in wildlife, then 5 cases in 2019, including 4 wildlife cases. Although 12 cases were detected in Poland in 2020 (6 among wildlife and 6 among domestic animals), the rabies situation in Poland greatly deteriorated in 2021. Rabies was reported in the central part of Mazowieckie voivodeship, Poland (*2*), a region that had been free of sylvatic rabies, for 17 years (*2*). The outbreak escalated through the end of 2021; a total of 113 cases were reported, 103 among wildlife (mainly foxes), and 10 among domestic animals (Figure 1). Isolate analysis revealed that all the cases were caused by a single Central Europe (CE) RABV variant (*2*) (Figure 2). Poland implemented ORV campaigns that controlled the outbreak, and continued surveillance detected no cases after 2022.

**Figure 1:**
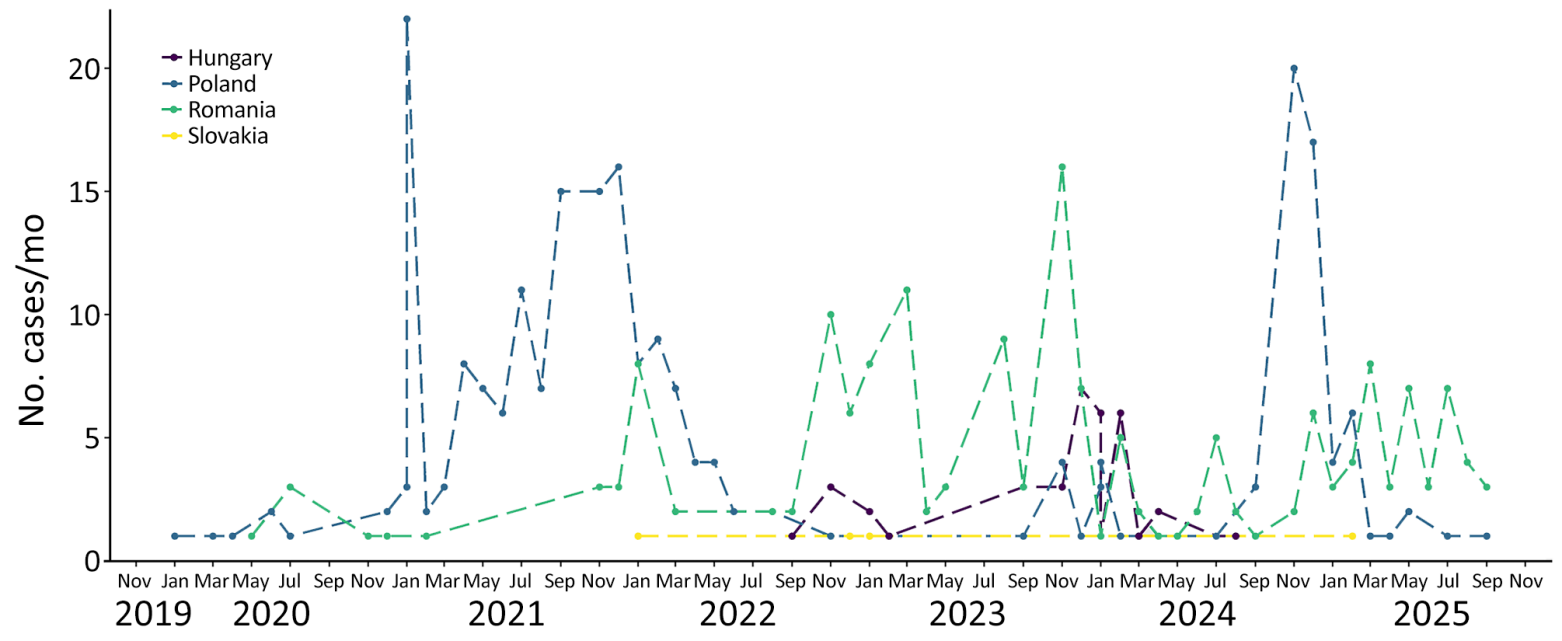
Distribution of detected rabies cases in wild and domestic animals from study of rabies reemergence, Central Europe, 2022–2024. The graph shows increases in rabies cases in Hungary, Poland, Romania, and Slovakia from the end of 2019 through September 2025.

**Figure 2:**
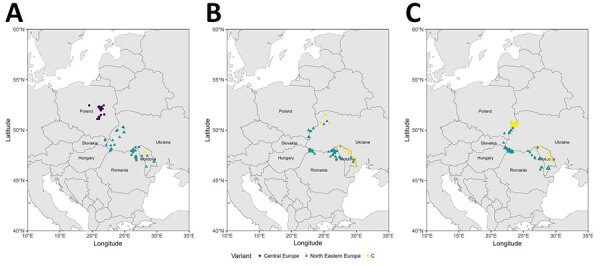
Spatial evolution of the different virus variants identified in a study of rabies reemergence, Central Europe, 2022–2024. A) 2022; B) 2023; C) 2024. For Ukraine and Moldova, only a few cases detected on their western borders have been sequenced; not all positive cases are noted.

A second major outbreak occurred in Central Europe in 2022, affecting Romania, Hungary, and Slovakia, then another in Poland in 2023 (*3*). Hungary and Slovakia had maintained a rabies-free status (according to the World Organisation for Animal Health self‑declaration of rabies‑free status that comply with Article 8.14.2 or 8.14.4 of the Terrestrial Animal Health Code) for several years but saw a resurgence of the disease in 2022, mainly near the eastern borders with Ukraine (*3*). During that outbreak, the North Eastern Europe (NEE) variant, previously detected in Poland, Slovakia, and Baltic countries (*4*), was identified. At that time, no recently published data identified variants circulating in Russia, Belarus, or Ukraine, making it difficult to compare variants and follow the geographic evolution of rabies. We analyzed RABV strains from Central Europe to assess migration of virus variants between countries during 2022–2024.

## The Study

We used rabies surveillance data from the Animal Disease Information System of the European Food Safety Commission (https://food.ec.europa.eu/animals/animal-diseases/animal-disease-information-system-adis_en#animal-disease-information) to document rabies case numbers. Since 2021, countries in the European Union have systematically identified RABV isolate strains by using Sanger sequencing of the full-length nucleoprotein (N) gene (1,353-bp) (Appendix 1), according to a method described in 2023 (*3*). 

Our phylogenetic analysis revealed the C variant in Poland in February 2024 and in Romania in August 2024 (Figure 2), although that variant is typically found in eastern Turkey, Georgia, Kazakhstan, Ukraine, and Russia (*4*,*5*). The number of related C variant cases then increased in Poland through the end of 2024, and we identified a single isolate from Romania in the same year. All other cases for which sequence data were available were the NEE variant.

The C variant includes viruses circulating in the steppe and forest-steppe regions of Russia from near its border with Europe to Tuva Province, as well as in Kazakhstan (*5*). The phylogeny shows that the C variant is spatially and genetically distinct from the NEE, CE, Eastern Europe (EE), and Western Europe (WE) variants (Appendix 2 Figure).

The NEE variant probably has the largest geographic range because that lineage has been detected in the Baltic region, Romania, Moldova, Poland, and Ukraine (*3*,*5**–**7*) and was the representative variant during the 2022 outbreak in Central Europe (*3*). The CE variant, initially isolated mainly in west and south Poland, eastern Germany, the Czech Republic, and Slovenia (*4*), was the variant involved in the 2021 outbreak in Poland (*2*). The EE variant has been shown to have a similar geographic distribution to the WE variant (Serbia, Bosnia and Herzegovina, Slovenia, Croatia, and Hungary) (*4*), and cases were also detected in northern Macedonia during 2011–2012 (*8*). The WE variant, previously detected in France, Italy, Slovenia, Croatia, Montenegro, and Bosnia and Herzegovina (*4*), was last reported during 2010–2011 (*9*), and could now be extinct.

A study of the full N gene from isolates collected during 2009–2022 from 13 regions of Russia near the border with Europe found that isolates belonged to variants C or D (*10*), as previously reported (*5*). That study also highlighted the predominance of C over D isolates and that variant C apparently replaced variant D in many regions (*10*). During 2002–2014, variant C was detected in the Baltic region (*11*), Ukraine (*12*), and Poland, which had 8 rabies cases along the border with Belarus during 2008–2014 (M. Smreczak, unpub. data). The C variant has not been previously isolated in Romania (*13*).

**Figure 3:**
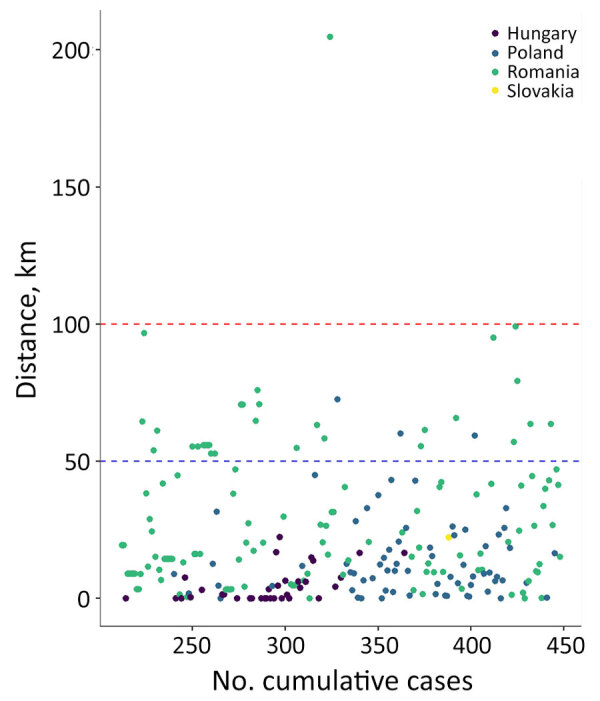
Distance from Ukraine and Moldova of cases detected in Hungary, Poland, Romania and Slovakia, January 2023–September 2025. Most cases were located <50 km from borders with Ukraine or Moldova (blue dashed line); almost all were <100 km from the border (red dashed line).

Using information on reported rabies cases, we calculated the distance to the nearest border for cases detected during January 2023–September 2025. Those calculations revealed that most rabies cases in Hungary, Poland, Romania, and Slovakia were located <50 km from borders with Ukraine or Moldova (mean 21.8, SD +24.9 km) (Figure 3). That distance appears to have remained unchanged over time, suggesting that the extent of the RABV infection might be stable in the region.

In Europe, the oral vaccination of wildlife reservoirs has proven to be the only effective measure to eliminate rabies (*14*). Since 2021, Western and Central Europe have experienced rabies challenges in the form of several distinct outbreaks, and the NEE variant has affected several countries at nearly the same time and remains active (*2*,*3*). The number of vaccinated regions in Ukraine has been increasing yearly since 2023, from 4 regions in 2023 to 14 in 2024 and 21 in 2025 (*15*). Although aerial ORV distribution is known to be more effective, manual distribution is used in Moldova and Ukraine because of local constraints. Given the rapid turnover of red fox populations, any interruption in ORV programs, as in Romania, likely will to lead to a drastic decline in vaccination coverage, which could enable RABV to spread more easily. Indeed, Romania reported an endemic human rabies case in 2025 caused by a free-roaming dog, but no endemic human cases had been reported in the European Union since 2012 (https://who-rabies-bulletin.org/news/tragic-human-rabies-case-romania). In 2024, two human rabies cases were notified in Ukraine, and 1 case was reported during the first quarter of 2025 (*15*). Those recent dramatic cases illustrate the need to redouble awareness and surveillance efforts to evaluate the infected areas and implement adequate ORV programs to avoid silent extension. Biologics can also be used as preventive measures, such as vaccinating humans and domestic species at risk when the reservoir is infected and untreated, or by administering postexposure prophylaxis.

## Conclusions

Through close and continuous cooperation between national reference laboratories for rabies and the extensive sequencing of the entire N gene of all rabies isolates since 2021, we detected the C variant and mapped its spread in Central Europe during 2024, reflecting a constant westward infection pressure and a fragile zoonotic situation. The ongoing war in the region is disrupting the organization of human societies and is affecting wildlife through the destruction of habitat and food resources. The effects of armed conflict on the spread of zoonoses, including rabies, should be assessed and given greater consideration. To overcome the worrying setback in rabies elimination in this part of Europe, regular, large-scale, and effective rabies vaccination campaigns need to be maintained and cross-border cooperation between countries needs to be strengthened.

Appendix 1 Sequences used in an investigation of rabies reemergence, Central Europe, 2022–2024.

Appendix 2 Phylogenetic tree of sequences in an investigation of rabies reemergence, Central Europe, 2022–2024.
