# Supplementary material for: Rabies Reemergence, Central Europe, 2022–2024
Source: Emerg Infect Dis. 2026 Feb;32(2):229–32. doi: 10.3201/eid3202.251597 (PMC12928247; doi:10.3201/eid3202.251597)
Supplement: Appendix 2 — Phylogenetic tree of sequences in an investigation of rabies reemergence, Central Europe, 2022–2024. [file 25-1597-Techapp-s2.pdf]

Article DOI: https://doi.org/10.3201/eid3202.251597

EID cannot ensure accessibility for supplementary materials supplied by authors. Readers who have difficulty accessing supplementary content should contact the authors for assistance.

Rabies Reemergence, Central Europe, 2022–2024

Appendix 2

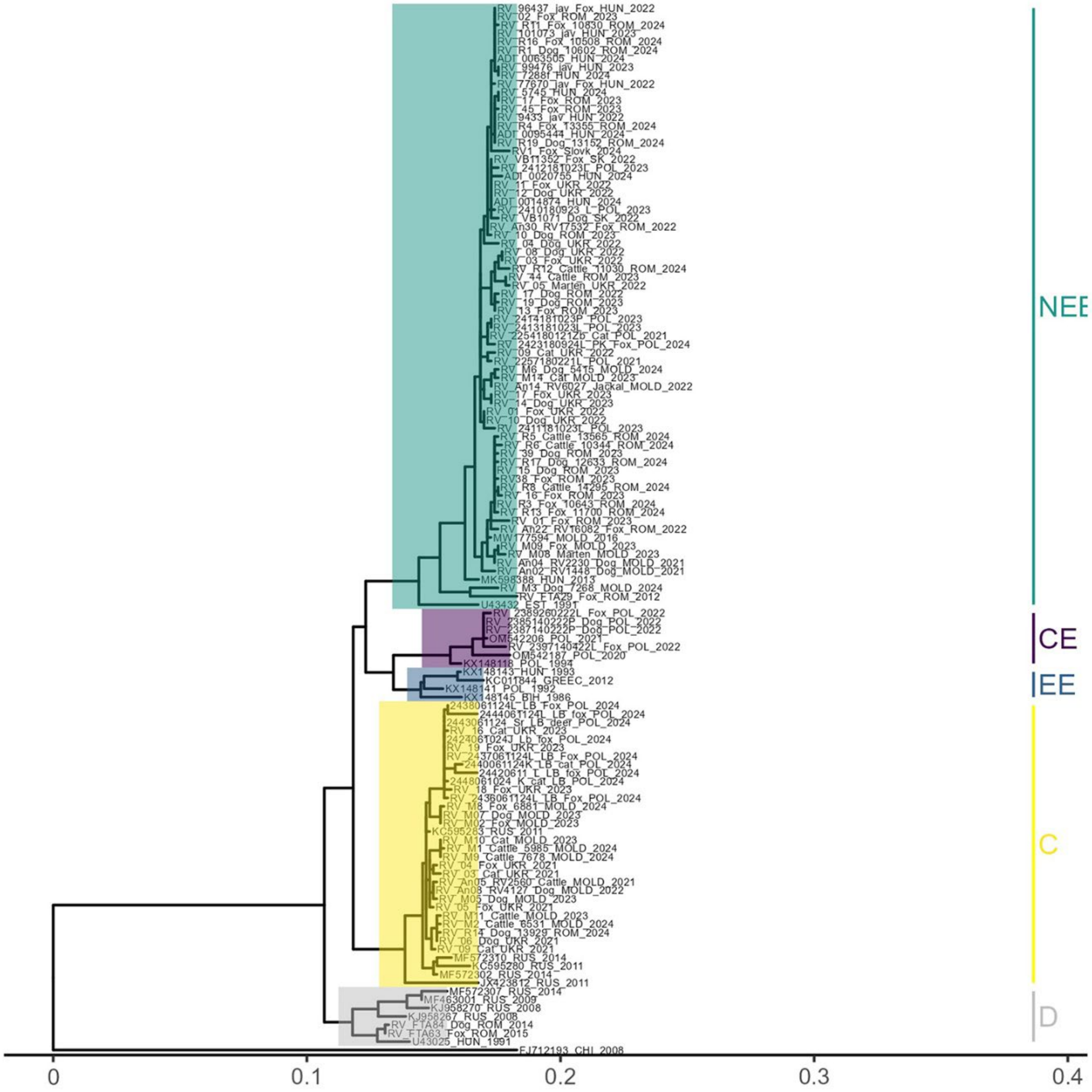

Appendix 2 Figure. Phylogenetic tree of rabies virus strains detected in Central Europe from 2022–2024. Colors correspond to those in Appendix 2 Table 2 (https://wwwnc/cdc/gov/EID/article/25-1597-App2.xlsx).
